# Soil properties rather than plant diversity mediate the response of soil bacterial community to N and P additions in an alpine meadow

**DOI:** 10.3389/fmicb.2022.1036451

**Published:** 2022-11-03

**Authors:** Zhenrong Lin, Lina Shi, Xiaoting Wei, Bing Han, Cuoji Peng, Zeying Yao, Qing Xiao, Xinmin Lu, Yanfang Deng, Huakun Zhou, Kesi Liu, Xinqing Shao

**Affiliations:** ^1^College of Grassland Science and Technology, China Agricultural University, Beijing, China; ^2^Institute of Ecological Protection and Restoration, Chinese Academy of Forestry, Beijing, China; ^3^College of Grassland Science, Gansu Agricultural University, Lanzhou, China; ^4^Tianshui Institute of Pomology, Tianshui, China; ^5^Qilian Mountain National Park Qinghai Service Guarantee Center, Xining, China; ^6^Key Laboratory of Restoration Ecology of Cold Area in Qinghai Province, Northwest Institute of Plateau Biology, Chinese Academy of Science, Xining, China

**Keywords:** plant diversity, soil bacterial diversity, soil properties, N and P additions, alpine meadow

## Abstract

The alpine meadow on the Qinghai-Tibetan Plateau, which is susceptible to global climate change and human activities, is subject to nutrient addition such as nitrogen (N) and phosphorus (P) to enhance soil available nutrients and ecosystem productivity. Soil bacterial community partly drivers the effects of nutrient additions on ecosystem processes, whereas the factors influencing N and P additions on bacterial community in alpine meadows are not well documented. We conducted a N and P addition experiment in an alpine meadow ecosystem on the Qinghai-Tibetan Plateau with four treatments: untreated control (CK), N addition (N), P addition (P), and NP addition (NP). We employed a high-throughput Illumina Miseq sequencing technology to investigate the response of soil bacterial community to short-term N and P additions. N and P additions decreased soil bacterial richness (OTU numbers and Chao 1 index), and P addition decreased soil bacterial diversity (Shannon and Simpson indices). N addition directly induced the change of soil $\text{N}{\text{H}}_{4}^{+}-\text{N}$, and decreased plant diversity. The N and P additions reduced soil bacterial community diversity, whose response was independent with plant diversity. Additionally, nutrient additions altered soil bacterial community composition, which were highly correlated with soil properties (i.e. pH, $\text{N}{\text{H}}_{4}^{+}-\text{N}$, and TP) as shown by RDA. Consistently, structural equation modeling results revealed that N addition indirectly acted on soil bacterial community through altering soil available nutrients and pH, while P addition indirectly affected bacterial community by increasing soil P availability. These findings imply that more attention should be paid to soil properties in regulating belowground biodiversity process in alpine meadows under future environmental change scenario.

## Introduction

Nitrogen (N) and phosphorus (P) availability often constrain the growth and metabolism of plant and soil microorganisms in terrestrial ecosystems (Dijkstra et al., 2012; Yuan and Chen, 2015; Jiang et al., 2021). Supplies and inputs of N and P have been greatly affected by pre-industrial revolution and human activities (e.g., fossil combustion and excessive fertilizer applications) (Ma et al., 2016; Yan et al., 2022). Presently, global inputs of N and P have increased by 84.0 and 43.3%, respectively, relative to the input before the 20th century (Peñuelas et al., 2013). Unbalanced N and P inputs are exacerbating P limitation or N and P co-limitation in terrestrial ecosystems, which generates drastic adverse effects on organisms performance (e.g., biodiversity loss) (Hooper et al., 2012) and weakens related ecosystem functions and services (Isbell et al., 2013; Spohn and Schleuss, 2019).

Soil microorganisms are important biotic factors in maintaining plant diversity and determining nutrient cycling (Jiang et al., 2019; Yang et al., 2021). For instance, soil bacteria regulate numerous ecological processes (Delgado-Baquerizo et al., 2017). In the past few decades, an increasing interest has been focused on the influence of N inputs on the soil bacterial community. N enrichment-induced loss of species diversity has been demonstrated to pose threats to both ecosystem stabilization and interactions between above and below ground (Geisseler and Scow, 2014). Previous studies have found that N fertilizer applications reduce soil bacterial diversity and alter the bacterial community composition (Dong et al., 2020; Sui et al., 2022). Ramirez et al. (2012) found in an incubated experiment that consistent N addition altered soil bacterial community composition with the increase in the relative abundance of *Actinobacteria* and *Firmicutes* and the reduction in the relative abundance of *Acidobacteria* and *Verrucomicrobia*. However, soil bacterial community composition is inconsistently responsive to N additions, possibly due to ecosystem-specific factors or variations in the soil nutrient status, plant community, and climatic factors (Yan et al., 2022).

P addition is paying much attention as a key determinant of the soil bacterial community (Ling et al., 2017; Long et al., 2018). However, there are still inconsistent results about the influence of P addition on soil bacterial community. For instance, in a Tibetan meadow, P input have been found almost no effect on the bacterial community composition (He et al., 2016); in a N-rich forest, P addition alters the diversity of soil bacteria, but do not affect the bacterial richness (Wang et al., 2018a); in a temperate meadow, the positive impact of P addition on soil bacterial richness is observed (Yan et al., 2022). These inconsistent results might be attributed to variation in ecosystem types and local environmental factors. Hence, a comprehensive understanding of the feedbacks in soil bacterial community to N and P additions is essential for evaluating the effects of global change and human activities on terrestrial ecosystem processes.

Nutrient additions can substantially alter soil bacterial community through direct and indirect effects on soil and plant properties (Zeng et al., 2016). N inputs indirectly alter soil bacterial community by affecting pH, resulting in a bacterial niche differentiation in semi-arid steppe (Ling et al., 2017). According to Ramirez et al. (2010), the response of soil microbial respiration to N addition is consistent irrespective of soil type and N form due to direct changes in N availability. The effect of change in soil available nutrient induced by N input on bacterial diversity and richness is also observed (Dimitriu and Grayston, 2009; Yan et al., 2018). Changes in soil P availability following P addition have been shown to be a key environmental factor determining soil bacterial community (Ling et al., 2017). A study conducted by Turner and Joseph Wright (2014) in a tropical forest demonstrated that P addition decreased microbial biomass by reducing soil phosphatase activity. The correlation between plant parameters and microbial community composition has been verified in grassland ecosystems (Sugiyama et al., 2008; Prober et al., 2015). The change of plant community resulted from nutrient inputs also affects soil bacterial community (Raynaud et al., 2021). Wei et al. (2013) demonstrated that N deposition weakened the effect of plant community on microbial community composition in a semiarid grassland. Hence, exploring how soil and plant characteristics affect soil bacterial community can improve our understanding of nutrient-induced ecological processes of ecosystems.

As the major ecosystem on the Qinghai-Tibetan Plateau (QTP), grassland ecosystems account for 68% of the total land area of QTP and about one-fourth of the total natural grassland area of China (Gai et al., 2009). The alpine meadow accounts for 35% of the total grassland area in QTP (Cao et al., 2004). The average rate of N deposition in QTP is up to 10 kg ha^−1^ yr^−1^ (Zhang et al., 2018). Furthermore, N and P fertilizers are usually applied to enhance soil available nutrients and the productivity of alpine meadow ecosystems (Yang et al., 2011; He et al., 2016). However, the effects of N and P additions on soil bacterial community composition were not well documented in Tibetan meadows. To evaluate the effects of short-term N, P and NP additions on the bacterial community composition of soil, we carried out a full-factorial N and P additions experiment in an alpine meadow. We hypothesized that: (1) N and P additions reduced soil bacterial diversity and changed community composition in the alpine meadow; (2) the responses of soil bacterial community to short-term N and P additions were regulated by the variation in soil properties and plant diversity.

## Materials and methods

### Experimental site

The experiment was conducted in a nutrient addition platform in an alpine meadow (36°55′*N*, 100°57′*E*, 3, 000~3,100 m a.s.l.), which was located in Haiyan County, Qinghai Province, northeastern Qinghai-Tibetan Plateau, China. Highland continental climate is characteristic of this region. The amount of mean precipitation is 400 mm per year and mainly distributes in the growing season between June and August. The average annual temperature is −0.45°C, with extremes of −29°C and 27°C distributed between January and July per year. The soil is clay-loam and classified as Mat-Gryic-Cambisol (Li et al., 2020). The main species of plants in this study area include *Leymus secalinus, Stipa purpurea, Poa pratensis, Kobresia humilis, Melissitus ruthenica, Potentilla chinensis*, and *Artemisia frigida*.

### Experimental design

The study area was fenced off to exclude large herbivores since 2019. The distribution of plants was fairly homogeneous, with few visible patches of individual species. This experiment was conducted using a completely randomized block experiment consisting of four treatments with four replicates. Each treatment was set up as following: the control plots with no fertilization (referred as CK), N fertilizer with a rate of 100 kg N ha^−1^ yr^−1^ (referred as N), P fertilizer with a rate of 80 kg P ha^−1^ yr^−1^ (referred as P), and the combination of N and P fertilizer at rates of 100 kg N ha^−1^ yr^−1^ and 80 kg P ha^−1^ yr^−1^ (referred as NP), respectively (Luo et al., 2019). N and P fertilizers were applied as forms of urea and superphosphate, respectively. After sunset, fertilizers were manually applied to the plot ground surface (for higher moisture levels) on early May, 2019, 2020, and 2021, respectively. Each experimental plot area was 3 m length × 4 m width. A 1 m width buffer strip was designed to avoid disturbance to neighboring plots as much as possible.

### Soil sampling and analysis

We collected soil samples from each plot of four treatments in August 2021. Each plot was sampled by taking five cylindrical soil cores (3.5 cm diameter and 10 cm depth) and mixing them thoroughly to form one composite sample (i.e., 16 composite samples). After collected, samples were put into self-sealing bags and immediately placed in a cool box and transported to the laboratory. In the lab, each soil sample was sieved (2 mm) to remove visible roots and little rocks. And then, they were separated into three sub-samples. And two of the three sub-samples were stored at −80°C until extraction of DNA and 4°C for soil water content and available nutrients analysis, respectively, while the remainder was air-dried for physico-chemical analysis.

Soil pH was measured using a pH probe (Thermo Fisher Scientific Inc., Beverly, MA, USA) in distilled water at a 1:2.5 (wt/vol) ratio. Soil available nitrogen ($\text{N}{\text{H}}_{4}^{+}-\text{N}$ and $\text{N}{\text{O}}_{3}^{-}-\text{N}$) were performed with continuous-flow analyzer as previously described (Han et al., 2021). Soil total phosphorus (TP) was determined colorimetrically at 880 nm by digesting samples and reacting them with molybdenum blue.

### Characterization of plant community composition

Plant community composition investigation and sampling were performed at the period of maximum sward biomass of August, 2021. Plant coverage was identified by visual estimation. Briefly, one quadrat (0.5 × 0.5 m) with 100 meanly distributed grid points was placed in each plot. Visual estimates of the relative coverage of each species were estimated in each quadrat. The Shannon-Wiener index (H) was employed to indicate plant diversity, which was calculated by the following Equation (Dong et al., 2020):


$$\begin{array}{l} H=\;-\overset{s}{\underset{i\;=\;1}{\sum }}\left(P_{i}lnP_{i}\right) \end{array}$$

where Pi represents the relative biomass of the i species of plant, and S represents the total number of species in each plot.

Aboveground part of plant species was mowed to the ground level and then divided into three functional groups (grasses, legumes, and forbs). After that, the plant belowground biomass was obtained by taking three random soil cores (10 cm diameter, 0–20 cm depth) from each plot and washing them under running water on a 2-mm sieve. The collected above and belowground parts were oven-dried at 65°C for 72 h, and then weighed.

### DNA extraction and quantification

Bacterial DNA extractions from 0.1-g of fresh soil were performed using the Omaga DNA Kit (Omega Bio-Tek, Norcross, GA, USA). Then, the extracted DNA samples was quantified by a spectrophotometer and the 1% agarose gel electrophoresis was used to assess DNA quality. The 16S rRNA gene V3-V4 region was amplified with the primer set 515F (5′-GTGCCAGCMGCCGCGGTAA-3′) and 907R (5′-CCGTCAATTCMTTTRAGTTT-3′). The PCR procedures were conducted according to the manufacturer's instructions. The raw sequence data were submitted to the Sequence Read Archive of the National Center for Biotechnology Information, USA (No. PRJNA884813).

### Bioinformatics for bacterial community characterization

We used QIIME2 (v2019.4) software platform to demultiplex the raw 16S RNA sequence data using the q2-demux plugin and then cut the primers using the cutadapt plugin (Martin, 2011). We then call the DADA2 pipeline via q2-dada2 to perform a series of quality control operations including de-priming, quality filtering, denoising, splicing and de-chimerisation (Callahan et al., 2016). Each de-duplicated sequence generated using DADA2 quality control was called ASVs (amplicon sequence variants). After that, the non-single ASVs were compared using MAFFT (Katoh et al., 2002) and further processed using FastTree2 to build a phylogenetic tree (Price et al., 2009). Using the classify-sklearn naive Bayes taxonomy classifier in feature-classifier plugin, taxonomies were assigned to ASVs classified. The QIIME2 diversity plugin was used to assess the diversity (Shannon-Wiener index and Simpson index) of the bacterial community.

### Statistical analysis

The effects on soil properties, plant parameters, and soil bacterial diversity of N addition, P addition, and their interaction were tested using a two-way ANOVA. After that, we employed the least significant difference test (LSD) (at *P* < 0.05) to examine the difference in soil, plants, and soil microbes among four treatments. Prior to running ANOVA, TP was log-transformed due to its not meeting the normality. The statistical analyses of ANOVA were modeled with SPSS 23.0 software (SPSS, Inc., Armonk, NY, USA). Principal coordinate analysis (PCoA) and permutational MANOVA (“adonis” function in vegan package) were used to assess the effect of each treatment on the soil bacterial community composition.

We conducted a redundancy analysis (RDA) to evaluate the correlation between soil bacterial community composition and soil parameters (pH, TP, and $\text{N}{\text{H}}_{4}^{+}-\text{N}$) and plant community variables (plant coverage, plant Shannon index). Only the vectors of soil parameters and plant community variables with variance inflation factors (VIFs) < 10 were taken into account. We used the “rda” function for model construction, “anova.cca” for significance testing of variables and axes, and “vif.cca” for variance inflation factor analysis. To estimate the importance of each explanatory variable, we used a hierarchical partitioning and a permutation test to derive significance at a level of *P* < 0.05. RDA analysis was conducted in R, version 4.0.5 (R Core Team, 2019), based on the “vegan” and “rdacca.hp” packages (Lai et al., 2022).

We conducted a structural equation modeling (SEM) to explore direct and indirect relationships among plant diversity, bacterial community, and soil properties under treatments. The bacterial community composition used in the SEM were represented using the first axis of PCoa analysis.The fitness of the model was evaluated using the chi-square test (0< χ^2^/df≤2 and *P* > 0.05), the root mean square error of approximation (RMSEA ≤ 0.05), the high goodness-of-fit index (GFI > 0.90) and the comparative fit index (CFI > 0.90) (Zhang et al., 2013). SEM analysis was constructed using AMOS 23.0 (SPSS Inc., Chicago, IL, USA). The critical level of significance for all statistical testing was set at *P* < 0.05.

## Results

### Effects of N and P additions on soil and plant parameters

**Figure 1:**
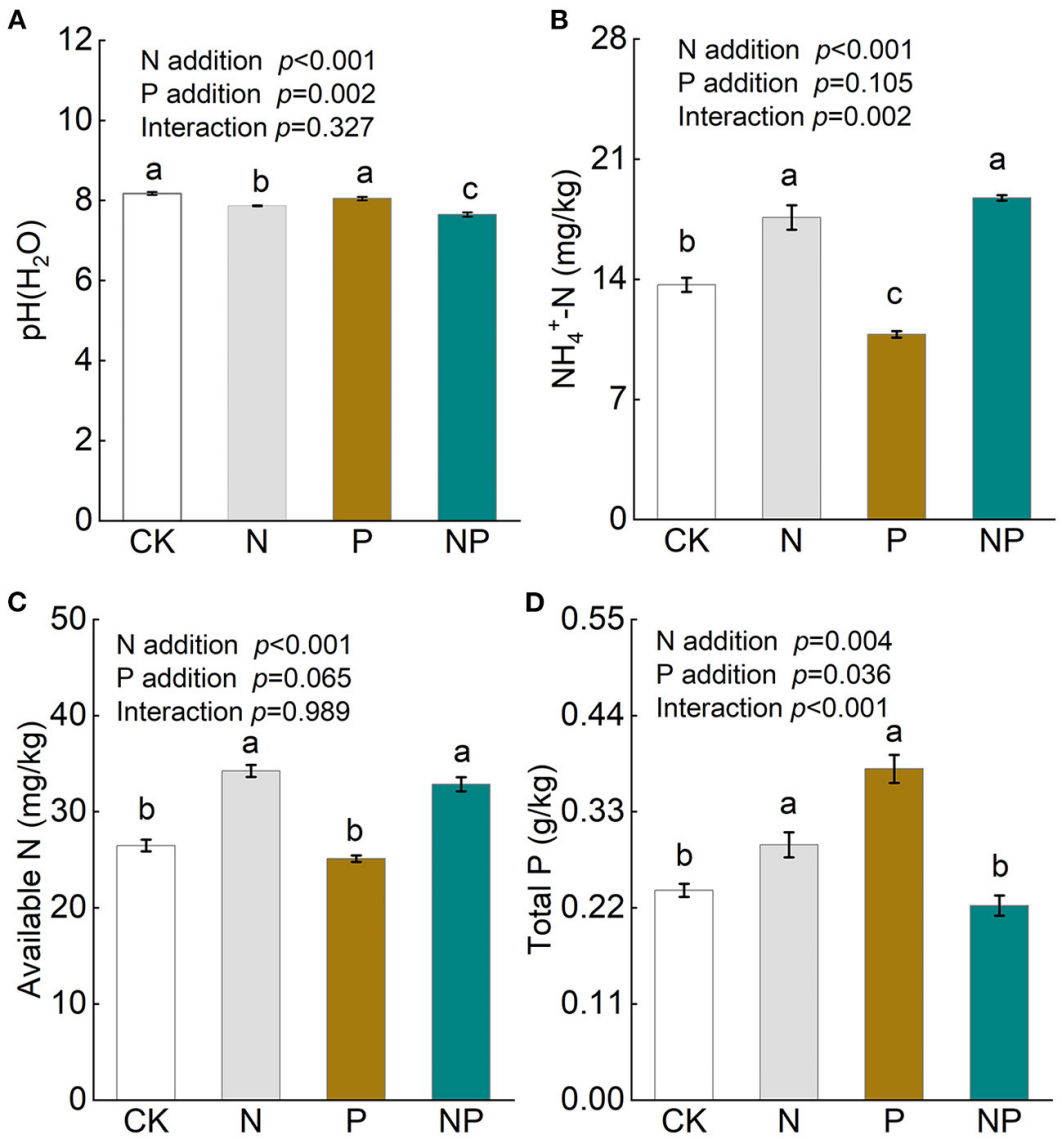
The variation of soil pH **(A)**, soil NH${}_{4}^{+}$-N **(B)**, soil available N **(C)**, and soil total P **(D)** under different nutrient additions. Values are means with SE (*n* = 4). Different lowercase letters represent significant difference among treatments determined by LSD-test (*P* = 0.05). CK, the control; N, nitrogen addition; P, phosphorous addition; NP, the combined addition of nitrogen and phosphorous.

Soil pH decreased significantly after the addition of N and NP, while it was unchanged by P addition (Figure 1A). Both N and NP addition increased soil available N, especially $\text{N}{\text{H}}_{4}^{+}-\text{N}$ concentrations, but were not significant between treatments (Figures 1B,C). P addition alone decreased soil $\text{N}{\text{H}}_{4}^{+}-\text{N}$ concentration (Figure 1B), while increased soil total P concentration (Figure 1D). Soil total P concentration was significantly increased by N addition but not NP addition (Figure 1D).

**Figure 2:**
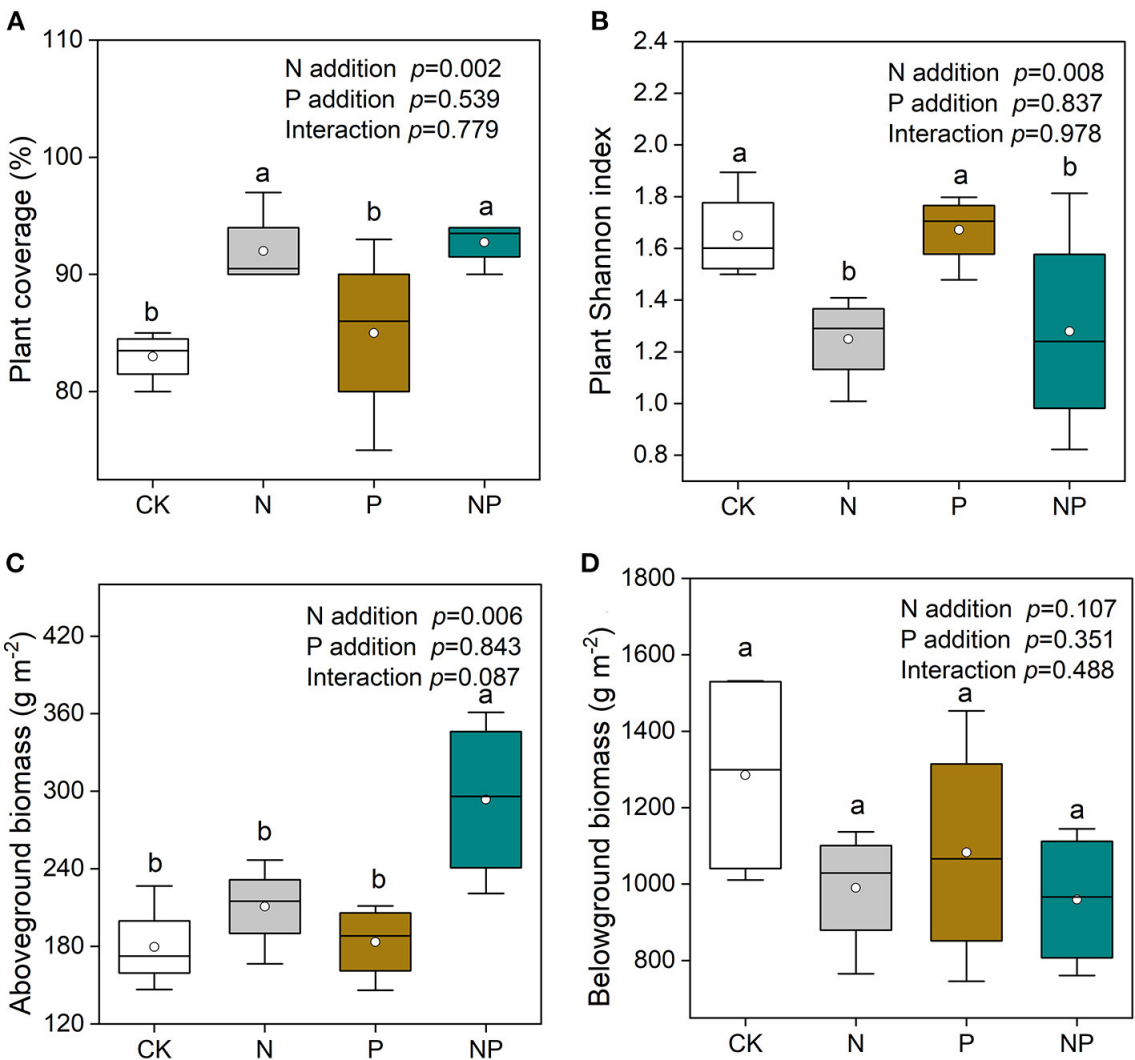
**(A)** The variation of plant coverage, **(B)** plant Shannon index, **(C)** total aboveground biomass, and **(D)** total belowground biomass under different nutrient additions. Values are means with SE (*n* = 0.4). Different lowercase letters represent significant difference among treatments determined by LSD-test (*P* = 0.05). CK, the control; N, nitrogen addition; P, phosphorous addition; NP, the combined addition of nitrogen and phosphorous.

N and NP addition significantly enhanced plant coverage and reduced plant Shannon index compared to CK and P addition (Figures 2A,B). N and NP addition significantly enhanced the relative aboveground biomass (AGB) of grasses (Supplementary Figure S1). Plant AGB was increased by NP addition compared to CK (Figure 2C). Total belowground biomass of plant was unchanged by N and P additions (Figure 2D).

### Effects of N and P additions on soil bacterial diversity

**Figure 3:**
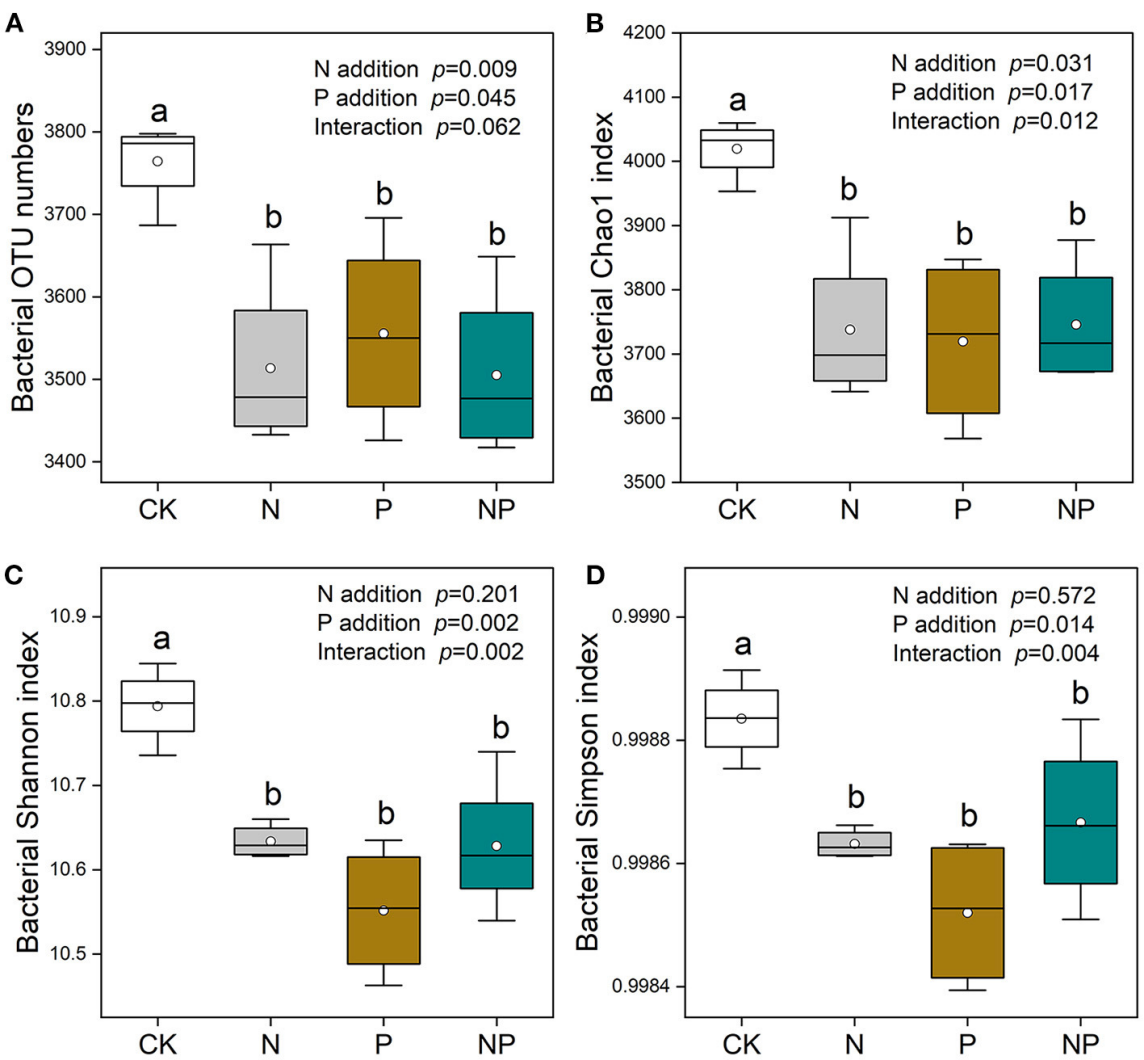
The variation of soil bacterial richness [OUT numbers **(A)** and Chao1 indices **(B)**] and diversity [Shannon **(C)** and Simpson **(D)** indices] under different nutrient additions. Values are means with SE (*n* = 4). Different lowercase letters represent significant difference among treatments determined by LSD-test (*P* = 0.05). CK, the control; N, nitrogen addition; P, phosphorous addition; NP, the combined addition of nitrogen and phosphorous.

Bacterial Chao1 richness and Shannon diversity rarefaction curves were saturated with enhanced sequence numbers (Supplementary Figure S2), indicating that the sequencing depth was sufficient. N and P additions significantly reduced bacterial richness (OTU numbers and Chao 1 indices). Lower bacterial OTU numbers and Chao1 indices were detected at the N, P, and NP addition treatments in comparison with CK (Figures 3A,B). The Shannon and Simpson indices of bacterial community were decreased significantly by N and P additions (Figures 3C,D).

### Effects of N and P additions on soil bacterial community composition

*Proteobacteria* (29.8%), *Actinobacteria* (28.9%), *Acidobacteria* (18.9%), and *Planctomycetes* (7.1%) were the predominant phyla in the soil bacterial community in all treatments (Supplementary Figure S3). The relative abundances of *Alphaproteobacterial* were significantly increased by P addition (*P* = 0.038), whereas the addition of N and NP did not act on the relative abundance of the four main bacterial taxa (Supplementary Figure S3). PCoA showed that bacterial community composition was obviously separated by treatments. The first two principal coordinates explained 33.26% of the total variation (PCoA 1 = 22.60%, PCoA 2 = 13.66%) in the bacterial community composition (Supplementary Figure S4). The bacterial community composition was shown to be statistically significant using PERMANOVA (Supplementary Table S1).

**Figure 4:**
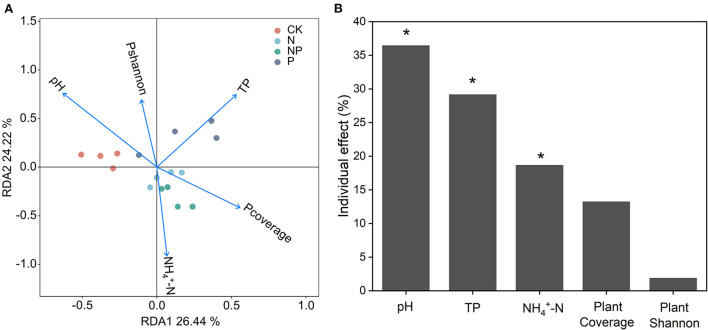
**(A)** Soil bacterial community composition and its variation partition ordination plot of a redundancy analysis (RDA) under N and P additions treatments. **(B)** The black bars for the independent explanation of bacterial community composition calculated by hierarchical partitioning. (The percentage of values shows adjusted R^2^, *indicates *P* < 0.05). TP, total phosphorus; $\text{N}{\text{H}}_{4}^{+}-\text{N}$, ammonium nitrogen; Pcoverage, plant coverage and Pshannon, plant Shannon. Proportions for the bacterial community composition were explained by soil properties and plant parameters.

Redundancy analysis (RDA) indicated that soil pH, TP, and $\text{N}{\text{H}}_{4}^{+}-\text{N}$ were the most significant explanatory variables for variation in soil bacterial community composition (Figure 4). There were 26.44% on the first constraint axis and 24.22% on the second constraint axis for all soil variables, which explained 50.66% of the total variation in bacterial community composition (Figure 4).

**Figure 5:**
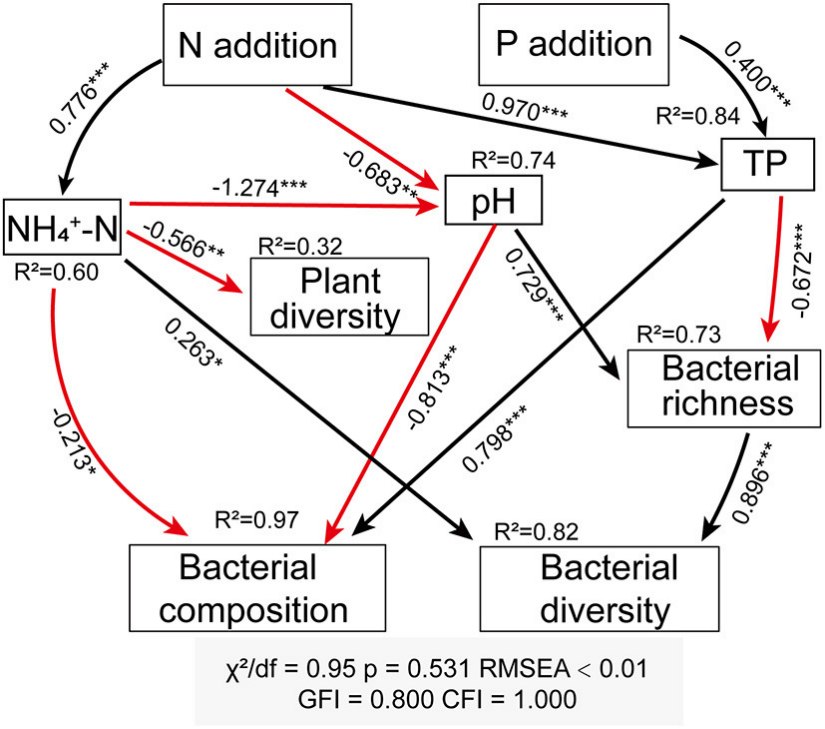
Structural equation modeling (SEM) analysis depict the regulatory pathway of N and P addition on plant diversity, bacterial composition, bacterial richness (Chao 1 index), and bacterial diversity (Shannon index). The solid black arrow lines represent positive effects, solid red arrow lines represent negative effects. Adjacent numbers labeled in the same direction as the arrow represent standardized path coefficients. Values of R^2^ indicate the proportion of variance explained by each variable in the model. *Indicates *P* < 0.05, **indicates *P* < 0.01, and ***indicates *P* < 0.001.

The structural equation modeling (SEM) revealed the integrated reaction of soil properties, plant parameters, and bacterial community to N and P additions. It provided a good fit between all variables (*P* = 0.53, χ^2^/df = 0.95, CFI = 1.000, GFI = 0.800, RMSEA <0.01), which accounted for 74% variation in pH, 84% in TP, 60% in $\text{N}{\text{H}}_{4}^{+}-\text{N}$, 32% in plant diversity, 97, 73, and 82% in bacterial composition, richness, and diversity, respectively (Figure 5). Overall, the increase in soil AN and the decrease in soil pH caused N and P additions were primary factors that contributed to the significant influence on the plant and soil bacterial community. N addition indirectly affected plant diversity through direct effect on soil $\text{N}{\text{H}}_{4}^{+}-\text{N}$. N and P additions decreased soil bacterial richness through affecting soil pH and TP, respectively. N addition had an indirect effect on bacterial composition through directly affecting $\text{N}{\text{H}}_{4}^{+}-\text{N}$, pH, and TP, while P addition indirectly affected soil bacterial composition through increasing soil TP. N addition indirectly affected bacterial diversity via mediating soil properties, and P addition reduced bacterial richness and indirectly regulated bacterial diversity (Figure 5).

## Discussion

Nutrient addition could affect soil bacterial community composition by directly or indirectly influencing plant diversity and soil properties (Dong et al., 2020; Chen et al., 2021). The significant response of soil bacterial diversity to N and P additions was observed in this study, suggesting that nutrient addition was an key influential factor (Klironomos et al., 2011; Ling et al., 2017). It is crucial to clarify the relationship among plant diversity, soil properties and bacterial community with regard to assessing the impact of nutrient addition on grassland ecosystem functions. The findings showed that soil bacterial community, including diversity and richness, was indirectly influenced by N and P additions through direct variation in soil available nutrients or acidification status. N and P additions had uncoupling effects on plant diversity and soil bacterial diversity. The results in an alpine meadow highlighted that soil properties, rather than plant diversity, regulated the response of soil bacterial community to short-term nutrient addition.

### Effect of N and P additions on bacterial community

In the present study, we found that N and P additions induced reductions in bacterial richness and diversity (Figure 3). N addition generated a significantly negative effect on bacterial diversity (Figures 3C,D), which was in accordance with previous studies (Zhou et al., 2015; Wang et al., 2018b). Soil pH can partially explain this phenomenon, and earlier studies found that the diversity of soil bacteria was affected by the decrease in soil pH following N addition (Roem et al., 2002; Wang et al., 2018a). As previously observed in tropical forests and temperate grasslands, bacteria richness decreased as enhancing N input (Zeng et al., 2016; Wang et al., 2018c). This supports our first hypothesis that N and P additions decreases soil bacterial richness. It is noteworthy that both P addition and the combination of N and P reduced soil bacterial diversity (Figures 3C,D), which highlighted that P was a growth-limiting nutrient for soil bacteria in an Tibetan alpine meadows. We observed that P addition significantly reduced the richness of soil bacteria (Figures 3A,B). It can be speculated that expanded P availability might exacerbate competition between soil bacteria and plants for nutrients, resulting in a reduction in soil bacterial richness following P addition (Zhang et al., 2014). In addition, enhanced soil available P resulting from P addition might reduce phosphate-solubilized bacteria, and thereby decreasing bacterial richness (Long et al., 2018). This result was conflicted with a non-significant effect of P addition on bacterial richness from old-growth tropical forests, which concluded that P addition failed to mitigate excessive N-derived soil acidification in an acidic environment (Wang et al., 2018c). These distinct phenomena might be attributed to the difference in ecosystem type, P application amount, and experimental duration.

*Proteobacterial, Actinobacteria, Acidobacteria*, and *Planctomycetes* were found to be the main bacterial phyla in alkaline meadow soil, which was in line with recent studies in an temperate meadow (Yan et al., 2022) and Tibetan alpine steppe (Dong et al., 2020). The RDA result demonstrated that the change in soil bacterial community composition was regulated by soil pH, TP, and $\text{N}{\text{H}}_{4}^{+}-\text{N}$ (Figure 4). This coincides with a previous study that was conducted by Zeng et al. (2016), in which they showed that soil $\text{N}{\text{H}}_{4}^{+}-\text{N}$ and pH were the important factors acting on soil bacterial community. A strong correlation between soil TP and bacterial community composition was also confirmed in SEM (Figure 5), which suggested that increased soil P is conducive to shape soil bacterial community composition in an alpine meadow (Yan et al., 2022).

### The relationship between soil and plant parameters and bacterial community composition and diversity following nutrient addition

As shown in SEM, soil parameters, but not plant diversity, had significant direct and indirect effects on bacterial diversity and richness (Figure 5), which partly supported our second hypothesis. Soil is a powerful ecological filter influencing the composition and diversity of soil bacterial community (Ling et al., 2017). Among the soil parameters mediating bacterial community, pH has been expected as a main factor (Lauber et al., 2009). N addition induced a substantial reduction in soil pH, while a non-significant effect of P addition was observed (Figure 5), which could be considered as a potential reason for the differential responses of bacterial richness and diversity to N and P additions. Soil acidification caused by N addition facilitates acidophilic bacterial growth, which might conversely inhibit the activity of other bacterial taxa (Zhang et al., 2014; Song et al., 2022). N addition reduced soil bacterial richness, which probably resulted from the fact that it narrows the optimal range of pH for bacteriological growth (Rousk et al., 2010; Husson, 2013). SEM also indicated that the declined pH caused a reduction in bacterial richness, and thus reduced bacterial diversity (Figure 5). The high-rate urea input (N input) can also cause ammonia toxicity to soil microbes (Vitousek et al., 1997; Geisseler and Scow, 2014), and then decreases soil bacterial richness. Furthermore, decreased soil pH and increased ammonium ionic strength caused by the high-rate N fertilizer application could be another potential reason for the decrease in bacterial richness (Demoling et al., 2007; Geisseler and Scow, 2014).

In line with recent findings (Zeng et al., 2016; Wang et al., 2018a), we observed that soil bacterial diversity was closely related to available N (Figure 5). Soil N availability, especially $\text{N}{\text{H}}_{4}^{+}-\text{N}$, is the preferred nutrient of soil bacteria (Nie et al., 2018). Most bacterial community, especially those in symbiosis with other organisms, maintain high abundances at the ammonium-rich environments (Müller et al., 2006). We also observed that $\text{N}{\text{H}}_{4}^{+}-\text{N}$ had significantly direct effects on bacterial composition (Figure 5), which was also supported by Zeng et al. (2016) and Wang et al. (2018b). It is well-documented that soil available N is an important factor in shaping the soil bacterial community. Furthermore, the findings demonstrated that the increase in TP due to P addition led to a reduction in soil bacterial richness (Figure 5), potentially because P addition exacerbates the competition between plant and soil bacteria for nutrients. The explanation of soil TP for a variation in bacterial richness was in agreement with a previous study observed in global grasslands, which suggesting that P element might be a limitation factor to bacterial richness (Prober et al., 2015).

Many studies have reported that there is no significant correlation between plant diversity and soil bacteria. For example, a study conducted in tallgrass prairie found that soil microbial diversity cannot be predicted by plant richness (Porazinska et al., 2003). In global temperate grasslands, the association between plant alpha diversity and soil bacterial community is not detected (Prober et al., 2015). Although plant diversity and bacterial diversity were affected by N and P additions, we did not find directly significant correlation (Figure 5). The results were inconsistent with previous studies, which found that soil bacterial diversity increased with enhancing plant diversity (Hiiesalu et al., 2014). The inconsistent results were probably attributed to asynchronous changes in plant and microbial diversity resulting from nutrient additions (Bardgett et al., 2005). The results also supported the view that plant diversity and soil bacterial diversity was largely uncorrelated.

## Conclusion

Soil properties directly acted on soil bacterial diversity and community composition, while plant diversity had no effect on bacterial diversity, following the short-term N and P additions in the alpine meadow. N addition directly caused an increase in soil $\text{N}{\text{H}}_{4}^{+}-\text{N}$, thus affecting the plant diversity. It indirectly modified the bacterial diversity and composition through altering available nutrients and pH, while P addition indirectly acted on the community by increasing P availability. Moreover, soil bacterial diversity was independent with plant diversity following nutrient additions. These results highlighted that the impacts of short-term nutrient addition on soil bacterial community were mediated by soil properties rather than plant diversity in alpine meadow ecosystems. Future studies should focus on the response of belowground biodiversity in alpine meadows under environmental change. This study provide evidence that soil microbial diversity responds to anthropogenic disturbances, which can help to understand soil belowground ecosystem processes and guide grassland management in future N and P enrichment scenarios.

## Data availability statement

The datasets presented in this study can be found in online repositories. The names of the repository/repositories and accession number(s) can be found in the article/Supplementary material.

## Author contributions

XS and KL conceived the study, supervised the writing, and revised the manuscript. ZL led the writing. LS, XW, BH, CP, ZY, QX, XL, YD, and KL contributed sections to the manuscript. HZ and XS provided funding support. All authors read and approved the final submission.

## Funding

This research was jointly supported by the National Natural Science Foundation of China (Grant Nos. 32171685 and 31971746) and the Second Batch of Forestry and Grassland Ecological Protection and Restoration Funds in 2020: Qilian Mountain National Park Qinghai Area Biodiversity Conservation Project (QHTX-2021-009).

## Conflict of interest

The authors declare that the research was conducted in the absence of any commercial or financial relationships that could be construed as a potential conflict of interest.

## Publisher's note

All claims expressed in this article are solely those of the authors and do not necessarily represent those of their affiliated organizations, or those of the publisher, the editors and the reviewers. Any product that may be evaluated in this article, or claim that may be made by its manufacturer, is not guaranteed or endorsed by the publisher.
