# Supplementary material for: Soil properties rather than plant diversity mediate the response of soil bacterial community to N and P additions in an alpine meadow
Source: Front Microbiol. 2022 Nov 3;13:1036451. doi: 10.3389/fmicb.2022.1036451 (PMC9668868; doi:10.3389/fmicb.2022.1036451)
Supplement: Supplementary file 1 [file Data_Sheet_1.docx]

| **Table. S1.** Results of PERMANOVA testing the effects of N and P additions on the soil bacterial community composition. | | | |
| --- | --- | --- | --- |
|  |  | pseudoF | P-value |
| All | - | 1.92 | **0.001** |
| CK | N | 1.95 | **0.035** |
| CK | P | 2.34 | **0.023** |
| CK | NP | 2.50 | **0.035** |
| N | P | 1.57 | **0.025** |
| N | NP | 1.48 | **0.028** |
| P | NP | 1.64 | **0.019** |


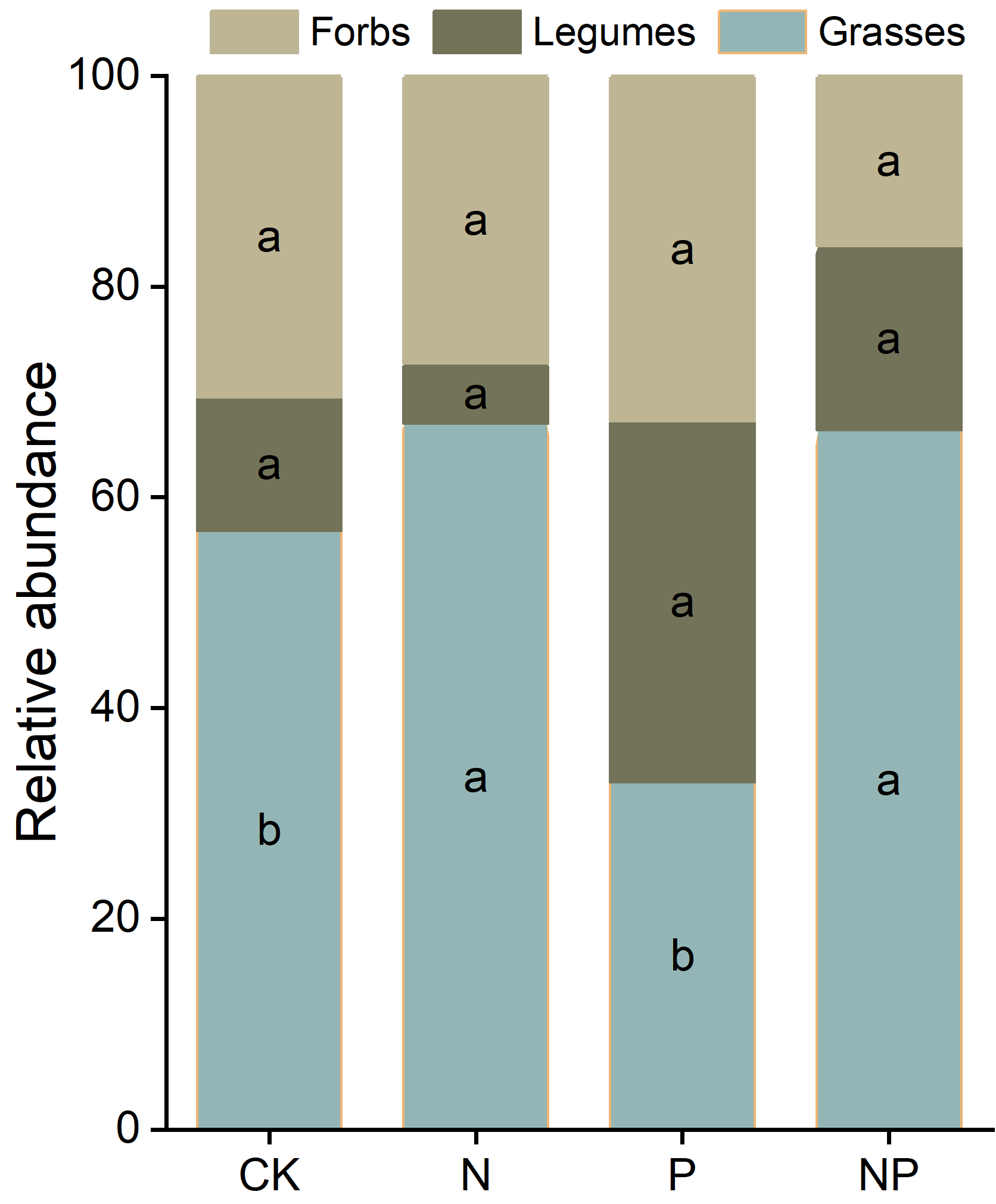


**Fig. S1.** Relative biomass of forbs, legumes, grasses under nutrient additions. Different lowercase letters represent significant difference among treatments. CK, the control; N, nitrogen addition; P, phosphorous addition; NP, the combined addition of nitrogen and phosphorous.

**
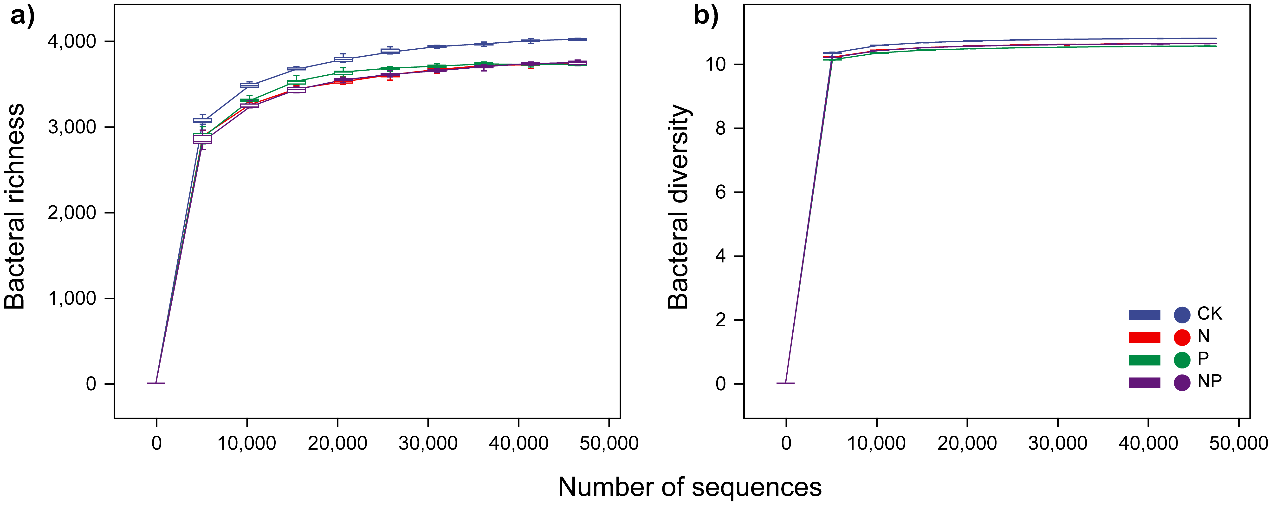
Fig. S2.** Rarefaction curves for soil bacterial richness (Chao1 index) and diversity (Shannon index) under different nutrient additions. CK, the control; N, nitrogen addition; P, phosphorous addition; NP, the combined addition of nitrogen and phosphorous.


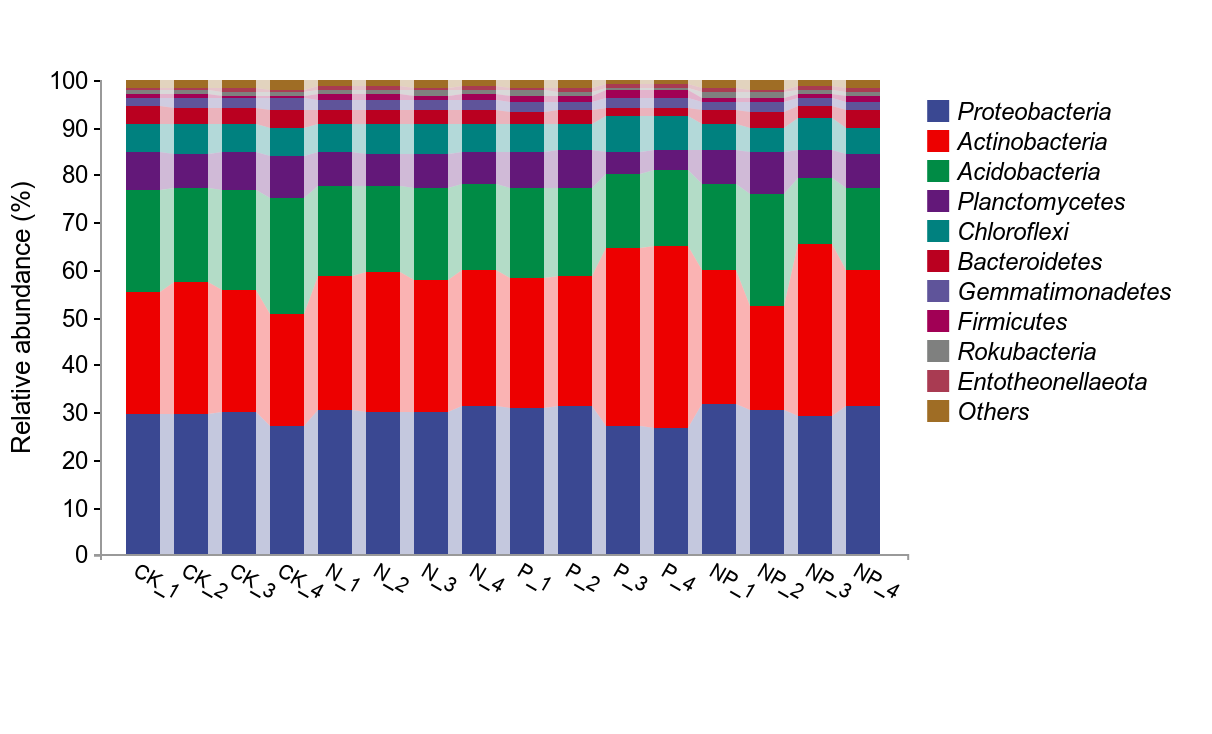


**Fig. S3.** Relative abundances of the soil bacterial taxa under nutrient additions. The top ten taxa in the relative abundance were shown, and those that were not in the top ten in the relative abundance were consolidated into "Others".


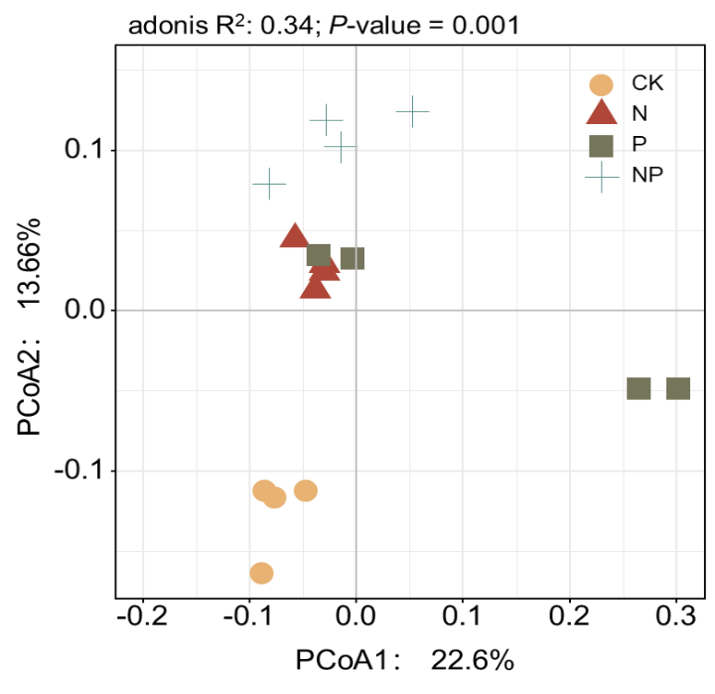


**Fig. S4** Bacterial community composition was assessed by principal coordinates analysis (PCoA) based on Bray–Curtis similarity distance. Each treatment is represented by a different color and shape. CK, the control; N, nitrogen addition; P, phosphorous addition; NP, the combined addition of nitrogen and phosphorous.
